# Relationship between SLAP Lesions and Shoulder Joint Capsule Thickness: An MR Arthrographic Study

**DOI:** 10.3390/medicina60081332

**Published:** 2024-08-16

**Authors:** Derya Güçlü, Veysel Uludağ, Mehmet Arıcan, Elif Nisa Ünlü, Hayri Oğul

**Affiliations:** 1 Department of Radiology, Faculty of Medicine, Duzce University, Düzce 81620, Turkey; nisaunlu@yahoo.com; 2 Department of Physiotherapy and Rehabilitation, Faculty of Health Sciences, Duzce University, Düzce 81620, Turkey; vuludag1365@outlook.com; 3 Department of Orthopaedics and Traumatology, Faculty of Medicine, Duzce University, Düzce 81620, Turkey; ari_can_mehmet@hotmail.com; 4 Department of Radiology, Medical Faculty, Medipol University, Istanbul 34083, Turkey

**Keywords:** glenoid labral pathology, shoulder joint capsule, MR arthrography, SLAP lesion

## Abstract

*Background and Objectives:* This study aimed to evaluate the relationship between SLAP lesions and the shoulder joint capsule thickness via MR arthrography. Understanding the relationship between SLAP lesions and the joint capsule thickness is important because an increased capsule thickness may indicate chronic inflammation and contribute to persistent pain and dysfunction. These findings have significant clinical implications for the diagnosis, management, and treatment strategies of shoulder joint pathologies. *Materials and Methods:* We retrospectively analyzed the MR arthrography results of 78 patients who underwent shoulder imaging at Düzce University Medical Faculty between October 2021 and November 2024. The study included patients diagnosed with SLAP lesions and compared them with a control group without such pathology. Data on joint capsule thickness at the level of the axillary recess, SLAP lesion type, cuff pathology, and demographic information were collected and analyzed. *Results:* The study included 32 patients with SLAP lesions and 46 control subjects. The mean age of the patients was 44.75 ± 14.18 years, whereas the control group had a mean age of 38.76 ± 13 years. The patient group presented a significantly greater mean anterior capsule thickness (3.13 ± 1.28 mm vs. 1.72 ± 0.7 mm, *p* = 0.0001), posterior capsule thickness (3.35 ± 1.32 mm vs. 1.95 ± 1.06 mm, *p* = 0.0001), and maximum capsule thickness (3.6 ± 1.32 mm vs. 2.06 ± 1.01 mm, *p* = 0.0001) in the axillary recess. SLAP type 2 lesions were the most common type (43.76%) in the patient group. *Conclusions:* This study revealed a significant association between SLAP lesions and an increased shoulder joint capsule thickness. These findings suggest that MR arthrography is an effective tool for assessing the joint capsule changes associated with labral tears, contributing to the better diagnosis and management of shoulder joint pathologies in clinical practice.

## 1. Introduction

The shoulder joint is one of the most mobile joints of the body and is therefore highly prone to injuries and pathologic changes [1]. Glenoid labral pathologies are among the common injuries that negatively affect the stability of the shoulder joint. The glenoid labrum is a cartilaginous structure surrounding the shoulder joint that plays a critical role in providing joint stability [2,3]. Labral tears or degenerative changes can be caused by trauma, repetitive use or other causes, and these conditions often lead to pain and a loss of function [4,5]. The joint capsule consists of connective tissue structures surrounding the shoulder joint and contributes to joint stability. The joint capsule thickness can be considered an indicator of inflammation, trauma or other pathologic processes in the joint [6]. The relationship between glenoid labral lesions and the joint capsule thickness has been explored in various studies [7,8]. For example, some studies have shown that labral tears can cause thickening or changes in the joint capsule. Labral tears can lead to mechanical instability in the shoulder joint, resulting in inflammation and the thickening of the joint capsule. This situation is associated with morphological changes in the connective tissue structures of the joint capsule due to repetitive stress and strain. Additionally, labral tears can affect the flexibility and function of the joint capsule, leading to restrictions on joint mobility and chronic pain. These pathological processes may manifest as significant thickening and structural changes in the joint capsule, which can be detected through clinical examination and imaging techniques [9,10]. This can have adverse effects on shoulder joint function and stability and cause clinical symptoms [11]. MR arthrography is a widely used imaging modality for the evaluation of shoulder joint pathologies. MR arthrography plays an important role in identifying pathologic changes in the glenoid labrum and joint capsule by visualizing intra-articular structures in detail. In the literature, MR arthrography has been shown to be an effective tool for evaluating the relationship between glenoid labral pathologies and the joint capsule thickness [12,13]. Numerous significant studies in the literature have examined the correlation between glenoid labral pathologies and the joint capsule thickness. For example, Kim et al. examined MR findings that predict shoulder stiffness in patients with full-layer rotator cuff tears. In this study, the joint capsule thickness and edema were found to be associated with shoulder stiffness [14]. Similarly, Park et al. emphasized the role of joint capsule abnormalities in the diagnosis of primary adhesive capsulitis and reported that the joint capsule thickness is associated with clinical symptoms [15]. However, studies directly examining the relationship between glenoid labral pathologies and the joint capsule thickness are limited in the literature. Most studies have focused on general findings related to shoulder stiffness and adhesive capsulitis [16,17]. Thus, research evaluating the specific relationship between superior labral anterior posterior (SLAP) lesions and the joint capsule thickness is lacking. The aim of this study was to evaluate the relationship between SLAP lesions and the shoulder joint capsule thickness via MR arthrography. The originality of this study lies in its direct examination of the specific relationship between SLAP lesions and the joint capsule thickness. Unlike existing studies in the literature, this research aims to determine how changes in the joint capsule are associated with SLAP lesions. These findings will contribute to a better understanding of shoulder joint pathologies and the development of treatment strategies.

## 2. Materials and Methods

### 2.1. Patients

This study is a retrospective archive review of patients who underwent shoulder MR arthrography at Düzce University Medical Faculty Hospital. After approval was obtained by the local ethics committee (1 July 2024, Decision No: 2024/144), patients who underwent MR arthrography of the shoulder between October 2021 and November 2024 were included in the study. The age, sex and clinical information of the patients was recorded using the hospital operating system. The inclusion criteria were patients in whom MR arthrography of the shoulder was performed at Düzce University Medical Faculty Hospital and those who were suitable for MR arthrography examination. The exclusion criteria included patients who had undergone shoulder surgery, acute trauma or the fracture of the shoulder, or those who had undergone a MR arthrography examination that was not sufficient to make appropriate measurements. Patients with primary adhesive capsulitis were excluded from the study. Patients who had not undergone surgery before, who did not have a rheumatological disease, and who underwent MR arthrography for any pathology other than a SLAP lesion were included as the control group. Furthermore, the diagnosis of SLAP lesions was established both radiologically and clinically. The clinical diagnosis was based on a combination of patient history, physical examination findings, and specific tests such as the O’Brien’s test and the crank test, corroborated by MR arthrography findings.

### 2.2. Injection Technique, MR Arthrograms and Image Analysis

**Figure 1:**
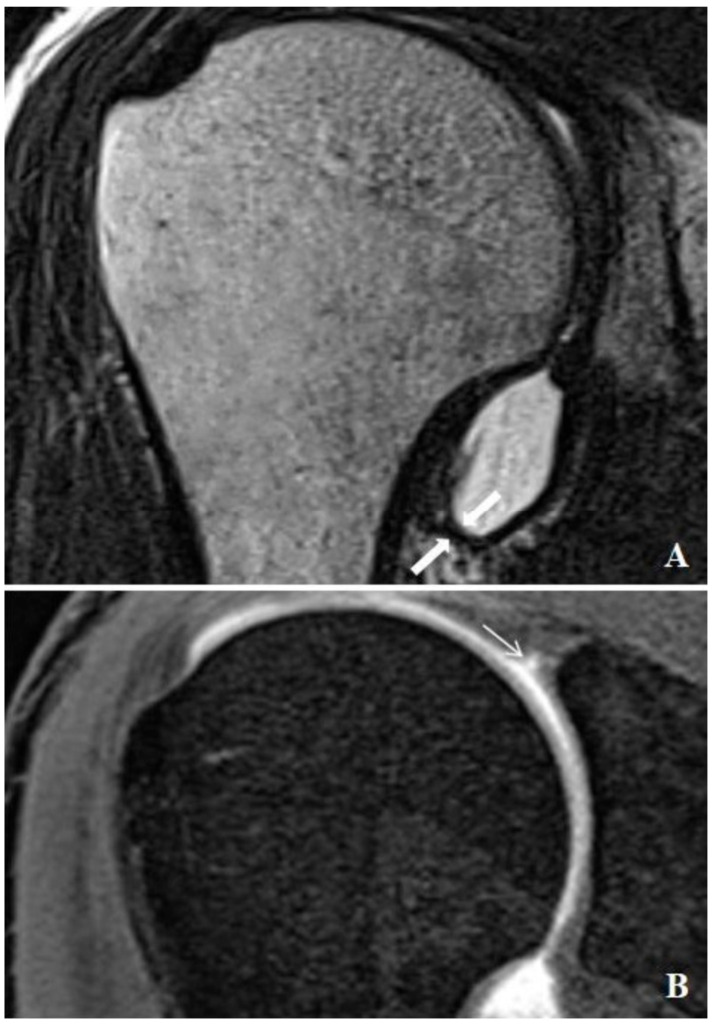
MR arthrographic examination of a 26-year-old male patient presenting with shoulder pain. (**A**) The thin-section nonfat-suppressed coronal oblique T2 image shows a thin joint capsule (thick arrow) at the level of the axillary recess. (**B**) Coronal oblique fat-suppressed 3D VIBE image of arthroscopically proven sublabral recess (thin arrow).

**Figure 2:**
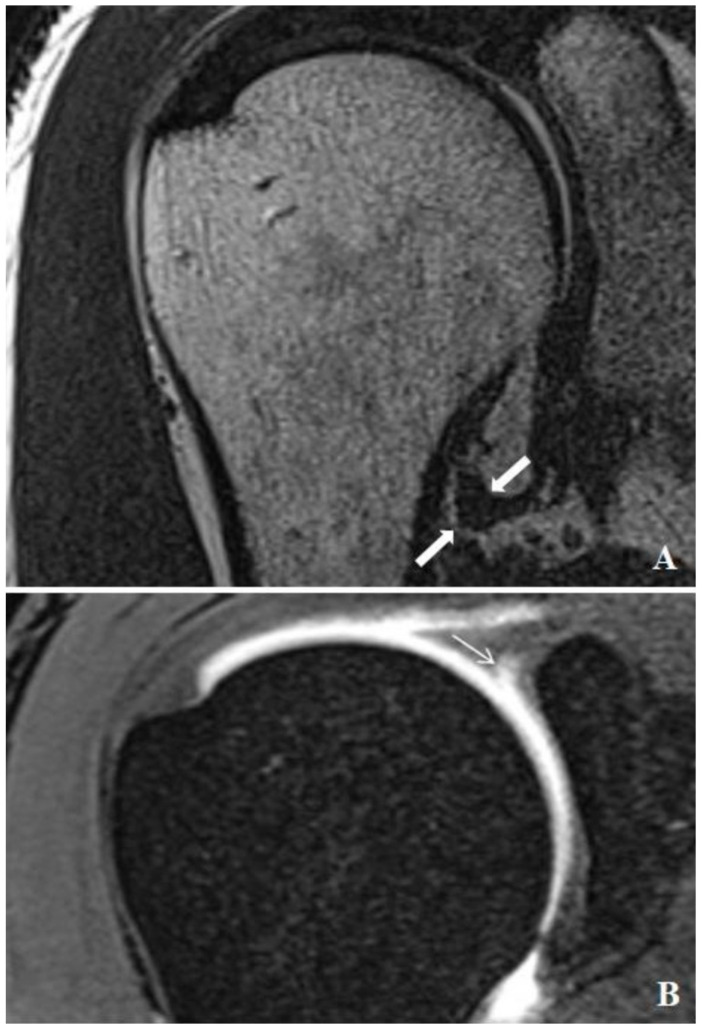
MR arthrographic examination of a 45-year-old male patient presenting with shoulder pain. (**A**) The thin-section nonfat-suppressed coronal oblique T2 image shows the marked thickening of the joint capsule (thick arrow) at the level of the axillary recess. (**B**) Coronal oblique fat-suppressed 3D VIBE image of arthroscopically proven SLAP type 2 lesion (thin arrow).

All injections were administered using a posterior approach under the guidance of ultrasound (GE Healthcare, Wauwatosa, WI, USA), without the use of sedation or premedication. A 20-gauge needle was utilized for the glenohumeral joint arthrography. Diluted contrast medium (0.1 mmol/kg, Gadovist, Bayer Schering Pharma AG, Berlin, Germany) was prepared at a 1:200 concentration and injected. The injection volume was adjusted based on the patient’s comfort level and tolerance. MR arthrography was performed 15–20 min post-injection using a 3T MRI system (Magnetom Skyra; Siemens Healthcare, Erlangen, Germany) with a dedicated shoulder coil. The imaging protocol included axial and coronal oblique fat-suppressed 2D T1-weighted TSE (TR/TE, 562/10; 3 mm slice thickness), sagittal oblique T1-weighted TSE (TR/TE, 725/10; 4 mm slice thickness), coronal oblique non-fat-suppressed thin section T2-weighted TSE (TR/TE, 4790/72; 0.6 mm slice thickness), and fat-suppressed T1-weighted 3D thin-section volume-scanning (VIBE) (TR/TE, 7.76/3.6; 0.4 mm section thickness). During data collection, MR arthrography images of the shoulder were reviewed on high-resolution monitors using a picture archiving and communication system (Syngo Via console, software ver. 2.0; Siemens Medical Solutions, Erlangen, Germany). The evaluation of the MR arthrography and measurement of the capsule thickness were conducted independently by two radiologists with 7 and 19 years of experience in musculoskeletal imaging. To ensure measurement consistency, inter-observer and intra-observer agreement tests were performed. The thickest part of the joint capsule was measured from both anterior and posterior aspects at the level of the axillary pouch using coronal T2 images (Figure 1A and Figure 2A). Measurements were taken from three consecutive sections, and the average value was calculated. Additionally, the thickest measurement of the joint capsule at the level of the axillary recess was recorded. The presence of rotator cuff tears, SLAP lesions, and the type of SLAP lesion were documented (Figure 1B and Figure 2B). This study compared a group of patients with SLAP lesions to a control group without such lesions, measuring various characteristics including age, sex, the presence of rotator cuff tears, the presence of SLAP lesions, and the type of SLAP lesion.

### 2.3. Statistical Analysis

In this study, statistical analyses were performed with the NCSS (Number Cruncher Statistical System) 2007 Statistical Software 07.1.21(Kaysville, UT, USA) package. In addition to descriptive statistical methods (mean, standard deviation, median, and interquartile range), the distribution of variables was examined with the Shapiro-Wilk normality test, an independent *t* test was used in the comparison of paired groups of normally distributed variables, the Mann-Whitney U test was used in the comparison of paired groups of variables that did not have a normal distribution, and the chi-square test was used in the comparison of qualitative data. The results were evaluated at a significance level of *p* < 0.05.

## 3. Results

The demographic and clinical characteristics of the control and patient groups are summarized in Table 1. The mean age of the control group was 38.76 ± 13 years, whereas the mean age of the patient group was 44.75 ± 14.18 years, with no statistically significant difference observed between the two groups (*p* = 0.058). The sex distribution also showed no statistically significant difference (*p* = 0.899), with 67.39% males in the control group and 68.75% males in the patient group. The side distributions were similar across the groups, with 47.83% of the right shoulders affected in the control group and 43.75% in the patient group (*p* = 0.722).

**Figure 3:**
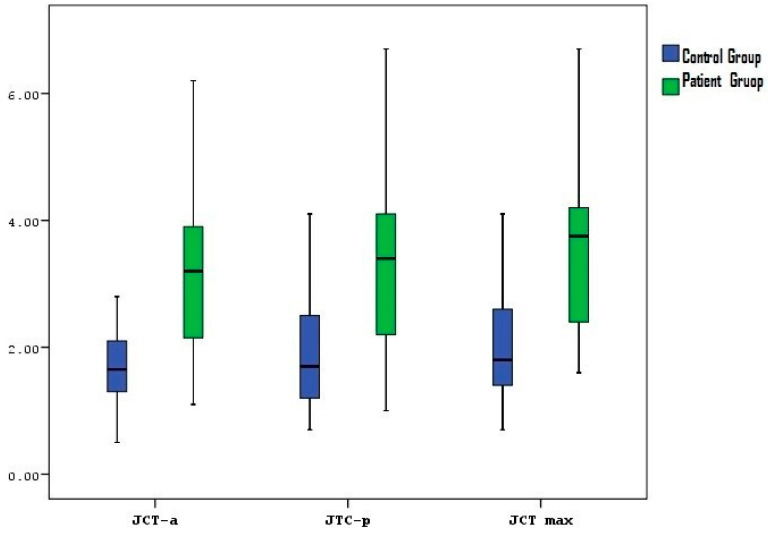
Distribution of the joint capsule thickness in the patient and control groups (joint capsule thickness—anterior: JCT-a, joint capsule thickness—posterior: JCT-p, joint capsule thickness—maximal: JCT-max).

The distribution of cuff pathology did not significantly differ between the control and patient groups (*p* = 0.116), with 23.91% of the control group and 40.63% of the patient group having cuff pathology. The mean anterior capsule thickness (joint capsule thickness–anterior (JCT-a)) in the patient group was significantly greater than that in the control group (*p* = 0.0001). Similarly, the mean posterior capsule thickness (joint capsule thickness-posterior (JCT-p)) and the thickest part of the joint capsule (JCT-max) were significantly greater in the patient group than in the control group (*p* = 0.0001 for both) (Table 1, Figure 3).

The distribution of the SLAP lesion types of the patient group is presented in Table 2. The most common SLAP lesion in the patient group was Type 2 (43.76%), followed by Type 1 (21.88%) and Type 5 (9.38%). The other SLAP lesions observed included Types 4, 8, 9, and 10.

## 4. Discussion

This study evaluated the relationship between SLAP lesions and the shoulder joint capsule thickness via MR arthrography. The mean symptom duration for patients with SLAP lesions was considerable. A prolonged symptom duration may lead to chronic inflammation and the thickening of the joint capsule. This relationship suggests that a longer symptom duration in SLAP patients could exacerbate capsular changes and contribute to persistent pain and dysfunction [18,19]. Studies in patients with full-layer rotator cuff tears have shown that the joint capsule thickness and edema are associated with shoulder stiffness. Information on the type of sports activities performed by patients in the SLAP group was collected, revealing a higher incidence among athletes involved in overhead and contact sports. Furthermore, the thickening of the posterior inferior glenohumeral ligament (posterior-IGHL) was noted in SLAP cases, which may be indicative of the ligament’s role in the pathophysiology of these lesions. Additionally, limitations on the external rotation range of motion and symptoms associated with adhesive capsulitis, such as pain and stiffness, were observed in our patient group. The MRI findings correlated with the clinical symptoms, indicating that an increased joint capsule thickness may contribute to these clinical presentations [20,21].

In the diagnosis of primary adhesive capsulitis, joint capsule abnormalities have been shown to be associated with clinical symptoms [22]. Our study makes an important contribution to the literature by examining the specific relationship between SLAP lesions and the joint capsule thickness. In particular, it provides more information about the effects of changes in the glenoid labrum and joint capsule on shoulder joint stability and function. It has also been reported in the literature that a longer preoperative symptom duration is associated with worse functional outcomes in patients with rotator cuff tears and shoulder stiffness [23]. This study suggests that changes in the joint capsule may be associated with the symptom duration and severity.

Furthermore, studies examining the correlation between MR arthrography and arthroscopy [24,25] have emphasized the importance of MR arthrography in the diagnosis of shoulder pathologies. Our study also revealed that MR arthrography is a reliable tool for the diagnosis of shoulder pathologies. Studies on the risk factors for and outcomes of shoulder stiffness after rotator cuff injuries [26,27] have shown that a high joint capsule thickness and edema in patients increase the risk of shoulder stiffness. This finding is consistent with the results of our study.

Our study provides a new perspective on the relationship between SLAP lesions and the joint capsule thickness. Furthermore, the examination of MR imaging biomarkers for clinical disorders and disease progression in patients with adhesive capsulitis of the shoulder suggests that MR imaging biomarkers are important in the evaluation of shoulder joint pathologies. Studies examining MR arthrography findings for the shoulder have also emphasized the importance of these methods in determining glenoid labrum damage [28]. In our study, the use of MR arthrography to evaluate the joint capsule thickness supports the effectiveness of such diagnostic methods.

In addition, the presence of cuff pathology was also greater in the patient group than in the control group, but this difference was not statistically significant. This finding emphasizes the importance of evaluating cuff pathologies together with SLAP lesions. Additionally, Ogul et al. found that extra-articular contrast material extravasations frequently occurred along the subscapularis muscle during shoulder MR arthrography, and that these extravasations were associated with adhesive capsulitis and SLAP lesions. These findings support the relationship between joint capsule thickening and the SLAP lesions observed in our study. Additionally, the presence of extravasations adjacent to the axillary recess does not always indicate glenohumeral ligament pathology, which aligns with our observation of an increased capsule thickness in SLAP lesion patients [29].

This study examined the relationship between SLAP lesions and the shoulder joint capsule thickness in detail and emphasized the effects of this relationship on clinical symptoms and shoulder joint function. Our findings may contribute to a better understanding of shoulder joint pathologies and the development of treatment strategies. The strengths of our study include its retrospective design, with a large cohort of patients, and the use of detailed imaging modalities such as MR arthrography. However, the study also has several limitations. Since this was a retrospective study, there may be some deficiencies in data collection. In addition, the fact that the study was conducted in a single center may limit the generalizability of the results. The lack of measures regarding interobserver agreement was one of our limitations. Another limitation is that arthroscopic correlation was missing in some patients. In future studies, prospective and multicenter studies are recommended.

## 5. Conclusions

In conclusion, this study examined the relationship between SLAP lesions and the shoulder joint capsule thickness in detail and emphasized the effects of this relationship on clinical symptoms and shoulder joint function. Our findings suggest that an increased capsule thickness is associated with SLAP lesions and may contribute to persistent pain and dysfunction. Understanding this relationship is crucial for improving the diagnosis, management, and treatment of shoulder joint pathologies. However, further research is needed to confirm these findings and explore the underlying mechanisms in greater detail. Future studies should include larger, multicenter cohorts and prospective designs to validate our results and provide more comprehensive insights into the clinical implications of SLAP lesions and the joint capsule thickness.

## Figures and Tables

**Table 1:** Demographic and clinical characteristics.

Characteristic.	Control Group (n = 46)	Patient Group (n = 32)	*p* Value
Age (Mean ± SD)	38.76 ± 13	44.75 ± 14.18	0.058 *
Gender			0.899 +
Male	31 (67.39%)	22 (68.75%)	
Female	15 (32.61%)	10 (31.25%)	
Side			0.722 +
Right	22 (47.83%)	14 (43.75%)	
Left	24 (52.17%)	18 (56.25%)	
SLAP Lesion			0.0001 +
None	46 (100%)	0 (0%)	
Present	0 (0%)	32 (100%)	
Cuff Pathology			0.116 +
None	35 (76.09%)	19 (59.38%)	
Present	11 (23.91%)	13 (40.63%)	
Joint capsule thickness- Anterior (Mean ± SD)	1.72 ± 0.7	3.13 ± 1.28	0.0001 †
Median (IQR)	1.65 (1.28–2.10)	3.20 (2.13–3.90)	
Joint capsule thickness- Posterior (Mean ± SD)	1.95 ± 1.06	3.35 ± 1.32	0.0001 †
Median (IQR)	1.70 (1.20–2.53)	3.40 (2.20–4.10)	
Joint capsule thickness- Max (Mean ± SD)	2.06 ± 1.01	3.6 ± 1.32	0.0001 †
Median (IQR)	1.80 (1.40–2.60)	3.75 (2.35–4.20)	

* Independent *t* test † Mann-Whitney U test + Chi-square test.

**Table 2:** SLAP lesion types.

SLAP Type	Patient Group (n = 32)
Type 1	7 (21.88%)
Type 2	14 (43.76%)
Type 4	3 (9.38%)
Type 5	3 (9.38%)
Type 8	3 (9.38%)
Type 9	1 (3.13%)
Type 10	1 (3.13%)

## Data Availability

The data is not publicly accessible due to privacy concerns but may be provided from the corresponding author upon request.
